# Mass-Spectrometric Identification of T-Kininogen I/Thiostatin as an Acute-Phase Inflammatory Protein Suppressed by Curcumin and Capsaicin

**DOI:** 10.1371/journal.pone.0107565

**Published:** 2014-10-09

**Authors:** Bina Joe, Anitha Nagaraju, Lalitha R. Gowda, Venkatesha Basrur, Belur R. Lokesh

**Affiliations:** 1 Department of Physiology and Pharmacology, University of Toledo College of Medicine, Toledo, Ohio, United States of America; 2 Department of Lipid Science and Traditional Foods, Central Food Technological Research Institute, Mysore, Karnataka, India; 3 Department of Protein Chemistry and Technology, Central Food Technological Research Institute, Mysore, Karnataka, India; 4 Department of Pathology, University of Michigan Medical School, Ann Arbor, Michigan, United States of America; University of Leuven, Rega Institute, Belgium

## Abstract

Curcumin and capsaicin are dietary xenobiotics with well-documented anti-inflammatory properties. Previously, the beneficial effect of these spice principles in lowering chronic inflammation was demonstrated using a rat experimental model for arthritis. The extent of lowering of arthritic index by the spice principles was associated with a significant shift in macrophage function favoring the reduction of pro-inflammatory molecules such as reactive oxygen species and production and release of anti-inflammatory metabolites of arachidonic acid. Beyond the cellular effects on macrophage function, oral administration of curcumin and capsaicin caused alterations in serum protein profiles of rats injected with adjuvant to develop arthritis. Specifically, a 72 kDa acidic glycoprotein, GpA72, which was elevated in pre-arthritic rats, was significantly lowered by feeding either curcumin or capsaicin to the rats. Employing the tandem mass spectrometric approach for direct sequencing of peptides, here we report the identification of GpA72 as T-kininogen I also known as Thiostatin. Since T-kininogen I is an early acute-phase protein, we additionally tested the efficiency of curcumin and capsaicin to mediate the inflammatory response in an acute phase model. The results demonstrate that curcumin and capsaicin lower the acute-phase inflammatory response, the molecular mechanism for which is, in part, mediated by pathways associated with the lowering of T-kininogen I.

## Introduction

Curcumin and capsaicin are naturally occurring phytochemicals that are present in two of the most widely used food additives in Asia, turmeric and hot peppers, respectively [1], [2]. Both of these dietary phenolic compounds have been extensively studied and found to possess significant health benefits as analgesics [3], [4], anti-tumor and anti-cancer agents [5], [6], antibiotics and also possess wound healing [7], [8], lipid-lowering [9], [10], anti-inflammatory [11], [12] and anti-arthritic properties [13], [14]. The mechanisms underlying these health effects have been extensively studied and reported [13], [15]–[29] and clinical studies are promising for the therapeutic potential of these dietary additives [30]. While there are notable differences between the two compounds for their reported mechanisms of action, common mechanisms are also reported, especially for their anti-inflammatory and anti-arthritic effects. For instance, both of these compounds significantly modify macrophage function by lowering the generation of the proinflammatory mediators, reactive oxygen species, metabolites of arachidonic acid, proteases and lysosomal enzymes [16], [31], [32].

In our previous studies, we demonstrated that both curcumin and capsaicin, when administered orally to rats with experimental arthritis, lowered their joint inflammation [33]. Given that the biological effects of curcumin and capsaicin are multiple and broad ranging from effects on transcription and translation of a variety of proteins, levels of lipids and epigenetic regulation [34], [35], we reasoned that while the anti-arthritic effect is partly attributed to the cellular effects of curcumin and capsaicin on macrophage function [16], [31], [32], there may be other potentially unexplored mechanisms that may additionally contribute to the observed lowering of joint inflammation in arthritic rats. To this end, we compared serum profiles of arthritic and normal rats. One prominent difference in the levels of a ∼72 kDa glycoprotein, GpA72, was observed between arthritic and normal rats [33]. GpA72 was significantly elevated in arthritic rats prior to the onset of arthritis and was therefore a useful biomarker predictive of future joint inflammation in rats. Notably, oral administration of curcumin and capsaicin lowered serum GpA72, which correlated well with the amelioration of arthritis [33]. Understanding the identity of GpA72 was therefore critical to determine a potentially novel mechanism of action underlying the anti-inflammatory property of curcumin and capsaicin. Accordingly, the goals of the current study were as follows: (I) To determine the identity of GpA72 by both by protein biochemistry and mass spectrometry based proteomic approaches and (II) Given that GpA72 appears during the acute phase of arthritis, assess whether GpA72 is an acute phase protein and study the ameliorative effects of curcumin and capsaicin on GpA72 under acute inflammatory conditions.

## Methods

### Ethics Statement

All animal experiments were conducted in accordance with approved protocols from the Institutional Animal Care and Use Committee of the Central Food Technological Research Institute (CFTRI), Karnataka, India.

### Purification of GpA72

Two methods were employed to purify GpA72 from chronic arthritic rats (I) The stained section of the gel corresponding to the region of GpA72 as per previously published procedures [33] was cut and placed in a dialysis bag with buffer Tris pH 6.5 and electroeluted [33]. (II) Sera from the experimental rats with acute joint inflammation were pooled and subjected to ammonium sulfate fractionation at 0–30%, 30–60% and 60–80%. The 30–60% fraction from ammonium sulfate precipitation containing GpA72 was suspended in 50 mM phosphate buffer pH 7.5 and loaded on to a Sephadex G-100 column equilibrated with the same buffer. Fractions were monitored at 280 nm. Further purification was by ion-exchange chromatography on a DEAE-Cellulose column. The bound proteins were eluted using 0.1 M to 0.5 M NaCl gradients in 25 mM TrisHCl buffer (pH 7.5). The fractions were analyzed on native PAGE. The fractions containing GpA72 were pooled and further purified by affinity chromatography on a concanavalin-Aagarose column. The bound glycoproteins were eluted with a linear gradient of α-D methyl mannopyrannoside in TBS buffer. The eluted proteins were screened for GpA72 by western blotting as previously described [33], pooled and concentrated by lyophilization. The purity of the protein was assessed by reverse phase HPLC. Total protein and sugar contents were determined by the Lowry method and the phenol sulfuric acid method as described previously [33].

### Amino Acid Composition

The GpA72 protein was hydrolyzed with 6N HCl under vacuum at 110°C for 24 h. Amino acid analysis was performed by pre-column derivatization with phenylisothiocynate. The phenylthiocarbomoylamino acids were analyzed using a Pico Tag column (3.9×150 mm) on a Waters HPLC system, equipped with a 1525 binary pump and Waters 2996-photodiode-array (PDA) detector set at 254 nm [36].

### Mass spectrometry

Coomassie stained bands corresponding to GpA72 were excised and subjected to trypsin digestion as described elsewhere [37]. Briefly, after reduction and alkylation of cysteines, the gel slices were incubated with sequencing grade trypsin overnight at 37 C. Peptides were extracted with 60% acetonitrile: 0.1% trifluroacetic acid. The extract was concentrated down to ∼15 ul. Two ul of the sample was separated on a reverse phase column (75 µm id ×5 cm×15 µm Aquasil C18 Picofrit column). The eluent peptides were directly sprayed onto an ion trap mass spectrometer (LCQ-Deca XP plus, Finnigan) equipped with a nanospray source. The mass spectrometer was operated on a double play mode where the instrument was set to acquire a full MS scan (400–2000 m/z) and a collision induced dissociation (CID) spectrum on the most abundant ion from the full MS scan. CID spectra were searched against R. norvegicus protein subset of SwissProt database using SEQUEST. Precursor (parent) mass tolerance was set to ±0.4 Da. Oxidation of Methionine (+15.99 Da) and carbamidomethylation of cysteine (+57.02 Da) were considered as variable modifications. All peptide identifications were manually verified.

### Induction of acute phase response in rats

Male, 70–100 g Wistar rats from the colony maintained at CFTRI, Mysore, were used for all experiments. To induce acute phase inflammatory response, hind limbs of rats (6 animals/group) were directly injected with one of the following proinflammatory agents - turpentine oil (250 µl), zymosan (500 µg suspended in sterile saline), carrageenan (500 µg in sterile saline), collagen (500 µg in 0.1 N acetic acid diluted with sterile saline) and attenuated cultures of mycobacterium tuberculosis H37Rv. The H37Rv cultures were delipidated with ether: alcohol (1∶1 v/v) thrice for 60 min each time. The delipidated material was air-dried and ground with a glass homogenizer and suspended to contain 5 mg/ml of liquid paraffin oil. 100 µl of this suspension was injected directly into the hind joints of ether anesthetized rats. Control rats received injections of liquid paraffin oil alone. Both control and experimental rats were allowed to recover from anesthesia and housed individually. Commercial food and water were provided *adlibitum*. Rats were euthanized on day 5 post-injection of acute-phase proinflammatory agents.

### Administration of anti-inflammatory compounds by gavage

Rats (n = 6/group) were either untreated (control group) or were given curcumin (30 mg/kg body weight) or capsaicin (5 mg/kg body weight) or piroxicam (10 mg/kg body weight) by gavage in groundnut oil once a day for 15 days prior to the administration of any attenuated cultures of mycobacterium tuberculosis H37Rv for induction of an acute phase response. Oral administration of anti-inflammatory compounds was continued until day 14, the day before euthanization. On day 15, when hind paw inflammation was clearly visible, all animals were euthanized.

### Serum Analysis

Immediately after euthanasia (on day 5 for acute phase studies and day 15 for arthritic rats), blood was drawn from these animals by cardiac puncture, allowed to clot and the separated serum was isolated by centrifugation at 2000 g for 15 min. The serum samples were diluted to 1∶50 with saline and further diluted 1∶1 with Polyacrylamide gel electrophoresis (PAGE) sample buffer (pH 6.8). The samples thus prepared were resolved by native PAGE on a 10% gel. The resolved proteins were stained with non-colloidal Coomassie blue.

## Results

### Purity of GpA72

**Figure 1:**
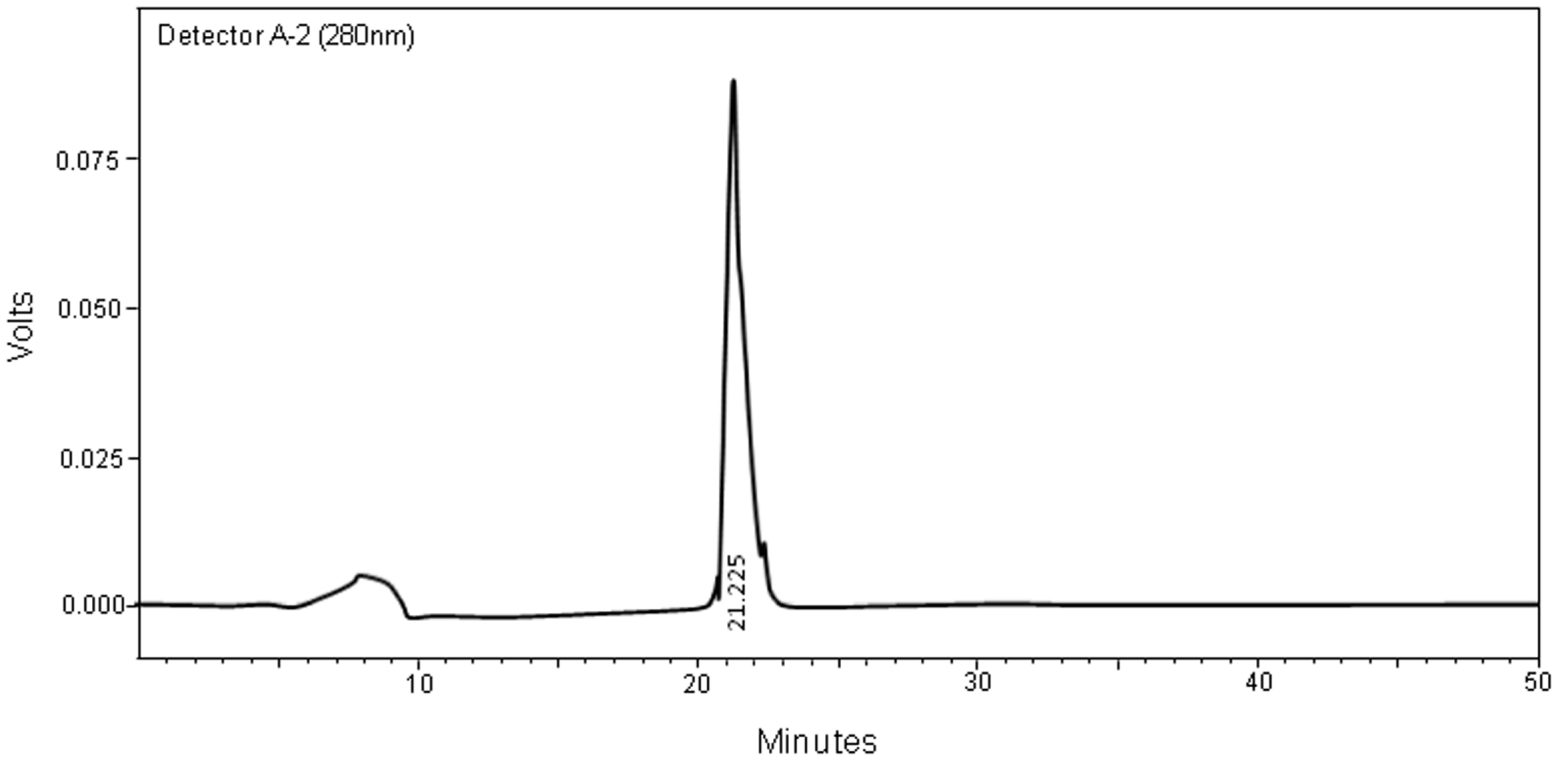
HPLC elution profile of the purified fraction of GpA72 Sera from four to six arthritic rats were pooled and subjected to protein purification as described under the methods section. The single peak eluting at 21.225 min represented GpA72.

Purification of GpA72 was undertaken by column chromatography as described under the Methods section. The HPLC profile of the purified GpA72 is shown in Figure 1. The single peak observed at the retention time of 21.225 min confirmed the purity of GpA72. This fraction was therefore used for further experiments.

### Amino acid composition of GpA72

**Figure 2:**
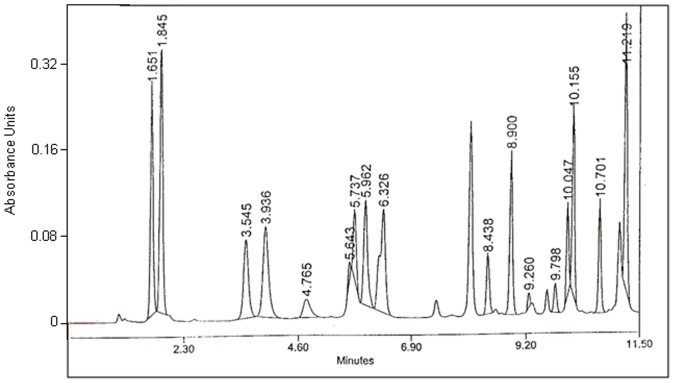
Amino acid content of GpA72. The protein was hydrolyzed with 6N HCl under vacuum at 110°C for 24 h. Amino acid analysis was performed by pre-column derivatization with phenylisothiocynate. The phenylthiocarbomoylamino acids were analyzed using a Pico Tag column (3.9×150 mm)on a Waters HPLC system, equipped with a 1525 binary pump and Waters 2996-photodiode-array (PDA) detector set at 254 nm. Numbers on the peaks are retention times in minutes.

The amino acid composition of the affinity purified protein showed that GpA72 was abundant in Aspartic acid (10%) and Glutamic acid (15%) (Figure 2 and Table 1). At 7–8%, Glycine, Tryptophan, Lysing, Alanine, Proline and serine contents were also high (Table 1). GpA72 had low contents of the sulfur containing amino acids, methionine and cysteine. It was also poor in basic amino acids such as Arginine and histidine (Table 1). Side-by-side comparison of the amino acid composition of GpA72 with that of α-Cystein protease inhibitor is shown in Table 1.

**Table 1:** Comparison of the amino acid compositions of GpA72 and α-Cysteine protease inhibitor.

Amino acid	GpA72 Mol %	α-Cysteine protease inhibitor Mol % (J. Biol. Chem. 1983, 258:12443-12447)
Asp	10.1	10.3
Glu	15.1	14.1
Gly	8.6	7.9
Thr	8	7.3
Leu	7.9	7.3
Pro	7.8	7.1
Ala	7.7	8
Ser	6.7	6.4
Val	6.5	4.7
Lys	5.5	*Not reported*
Ile	3.7	2.8
Phe	3.6	3.9
His	2.5	2.7
Tyr	2.5	2.3
Arg	1.7	2.9
Cys	1.5	4.1
Met	0.5	1.2

### Identification of GpA72 as rat T-kininogen I/Thiostatin

**Figure 3:**
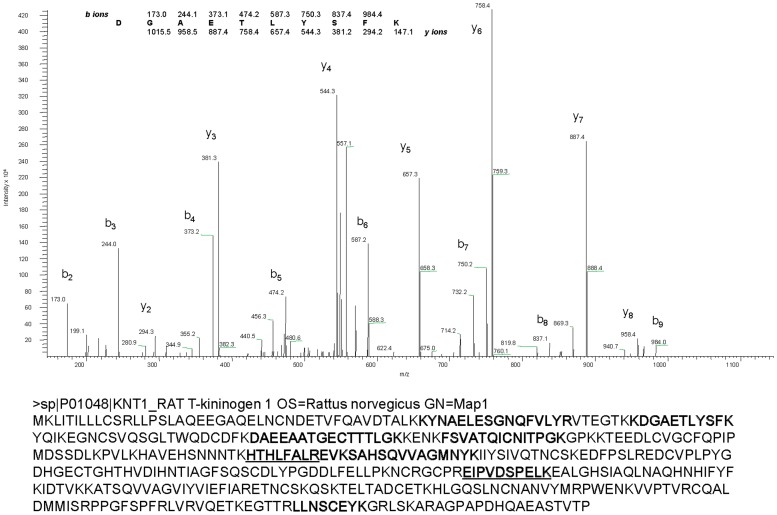
Identification of GpA72 as T-Kininogen I by tandem mass spectrometry analysis. Protein band corresponding to GpA72 was excised and protolysed with trypsin. Extracted peptides were analyzed by liquid chromatography Tandem mass spectrometry using an ion trap mass spectrometer (LCQ Deca XP, Finnigan) as described in the Methods section. Top panel shows CID spectrum that was matched to ^66^DGAETLYSFK^75^ of T-kininogen 1. Observed b- and y-ions are indicated. Whole protein sequence and the peptides identified by LC-Tandem MS (bold) are shown in the bottom panel. Peptide sequences identified that aid in distinguishing the T-Kininogen I from T-Kininogen II are underlined.

In-gel digestion followed by liquid chromatography tandem mass spectrometry (LC-MS/MS) was employed to identify GpA72 by direct peptide sequencing. Collision induced dissociation spectra (CID or MS/MS) were collected using an ion trap mass spectrometer. Searching CID spectra against rat proteome database identified several peptides matching to T-kininogen I variously known as Major Acute Phase protein and thiostatin (SwissProt Accession # P01048). A representative CID spectrum and all peptides identified are shown in Figure 3. No other proteins, except porcine trypsin (enzyme used for digestion) and keratins (common contaminant), were found in this analysis.

### Effect of Curcumin and Capsaicin on levels of T-kininogen I

**Figure 4:**
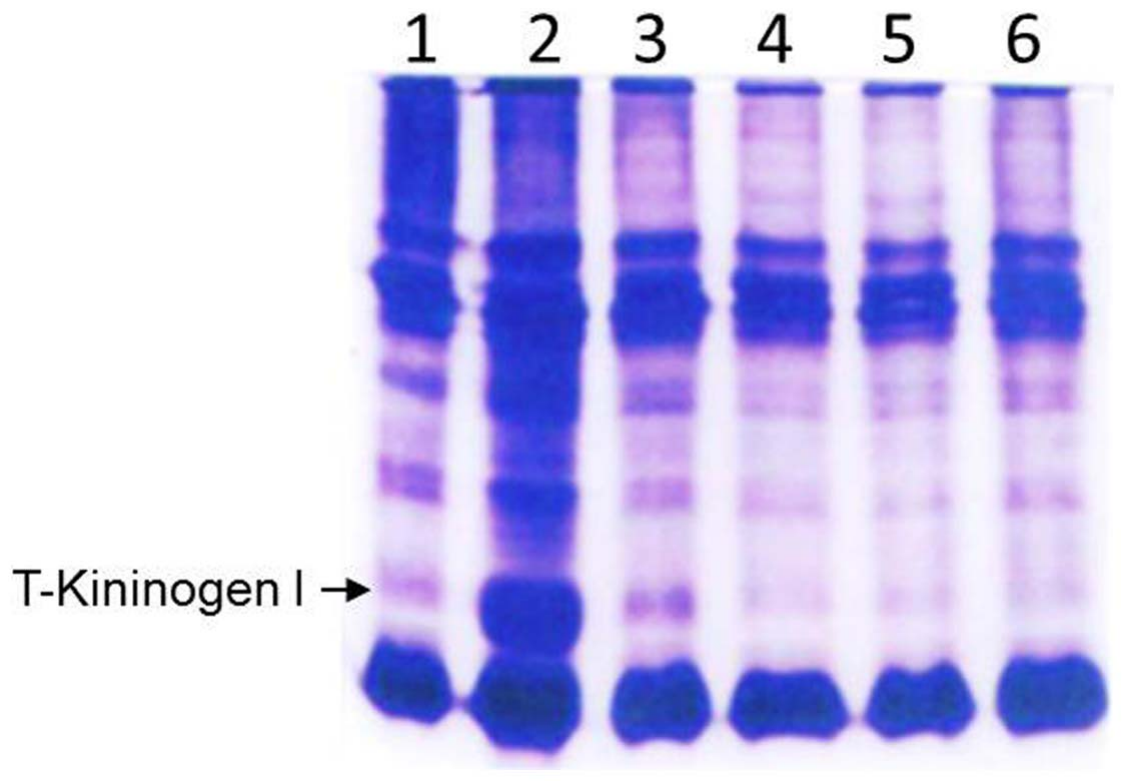
Serum levels of T-kininogen I in rats injected with different proinflammatory agents. Representative native PAGE gel image of sera from rats with hind joint injections of the following: Lane 1: Liquid paraffin oil, Lane 2: Adjuvant containing H37Rv, Lane 3: Turpentine oil; Lane 4: Zymosan; Lane 5: Carrageenan and Lane 6: Collagen.

The above findings on the identity of GpA72 as T-kininogen I, taken together with the previous demonstration of lowering of GpA72 in arthritic rats by the phenolic compounds, curcumin and capsaicin, suggest that oral administration of these compounds lower T-kininogen I levels in rats. Since T-kininogen I is an acute phase protein, we hypothesized that the beneficial effects of curcumin and capsaicin on chronic inflammatory conditions such as arthritis is mechanistically linked to the control of the acute phase of inflammation via modulation of levels of T-kininogen I. To test this hypothesis, we first tested whether other pro-inflammatory agents can also increase T-kininogen I. Hind limbs of rats were injected with known proinflammatory agents-turpentine oil, zymosan, carrageenan, collagen and adjuvant containing mycobacterium H37Rv. Increase in serum T-kininogen I was accompanied by severe joint inflammation in rats injected with adjuvant containing mycobacterium H37Rv (Figure 4). Rat injected with the other proinflammatory agents did not show an increase in serum levels of T-kininogen I (Figure 4) and also there was no inflammation in the joints of these animals with the exception of rats injected with turpentine oil which showed marginal increase in T-Kininogen I as well as a mild inflammatory response (Figure 4). Based on these observations, we chose the direct injection of adjuvant containing mycobacterium H37Rv as the acute-phase model to evaluate the effect of oral administration of curcumin and capsaicin on serum T-kininogen I levels.

**Figure 5:**
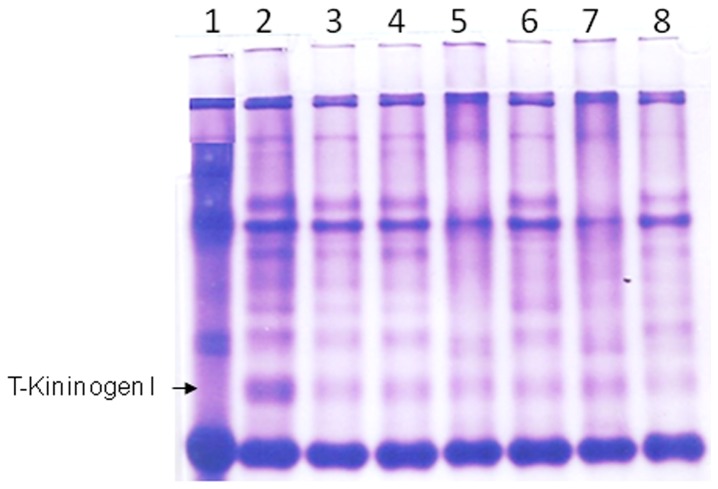
Lowering of T-Kininogen I levels by dietary curcumin and capsaicin. Representative native PAGE gel image of sera from rats with hind joint injections as follows: Lane 1: Liquid Paraffin oil, Lanes 2–8: Adjuvant containing H37Rv. Lanes 3–8 are serum samples from rats orally administered with various compounds as follows: Lanes 3 and 4: Curcumin; Lanes 5 and 6: Piroxicam, Lanes 7 and 8: Capsaicin.

As shown in Figure 5, rats that were injected with mycobacterial adjuvant alone, without oral administration of any anti-inflammatory agents had the highest increase of serum T-kininogen I (lane 2) compared with control rats that received the placebo (Lane 1). Compared to Lane 2 in Figure 5, rats administered orally with either curcumin or capsaicin demonstrated a significant reduction in their serum T-kininogen I levels. These lowering effects observed were comparable to the lowering effect of the known anti-inflammatory agent piroxicam.

## Discussion

Data from the current study clearly demonstrate the inhibitory potential of the two dietary compounds, curcumin and capsaicin, on serum levels of the acute inflammatory phase protein, T-kininogen I. The concurrent observation of lowering of joint inflammation in the rats fed with curcumin or capsaicin indicates that these compounds exert their anti-inflammatory effects by lowering serum levels of T-kininogen I.

Inflammation is part of the obligatory response of the body against infections from harmful pathogens. It is characterized by the four cardinal signs-pain, redness, swelling and heat [38]. Several biochemical pathways are well orchestrated to develop, sustain and remiss the inflammatory process as needed [39]. Dysfunctional regulation of the inflammatory response leads to chronic inflammatory and autoimmune diseases such as Rheumatoid Arthritis. Dietary anti-inflammatory agents have been observed to have significant beneficial effects in controlling the extent of inflammation in autoimmune diseases. Curcumin and capsaicin are two such dietary anti-inflammatory agents that have demonstrated beneficial influence on lowering chronic inflammatory responses. The current study demonstrates that in addition to the known anti-inflammatory effects of curcumin and capsaicin on the chronic inflammatory response, they also lower inflammation in the acute phase. The identification of T-kininogen I as the molecule lowered by the oral administration of curcumin or capsaicin suggests that these compounds participate in one of the characteristic events in the acute phase response to inflammation, which is a rapid rearrangement in the pattern of plasma protein concentrations. T-kininogens are plasma glycoproteins that were first identified as acute phase reactants in rat serum and later found to be cysteine proteinase inhibitors. In rats, there are four types of kininogens, two of which are classical high and low molecular weight kininogens and two of which are low molecular weight-like kininogens called ‘Third’ kininogens, designated as T-kininogenI and T-kininogenII. T-kininogenI and T-kininogen II are both 430 amino acid secreted rat proteins that each contain three cystatin domains and have nearly identical functions. Although peptide sequences obtained by mass spectrometry data clearly indicated that the 72 kDa glycoprotein lowered by curcumin and capsaicin is T-kininogen I, it is possible that some of the individual peptide sequences are also representing T-kininogen II. Nevertheless, the data points to the modulation of T-kininogens as a potential mechanism associated with the observed anti-inflammatory property of these dietary components.

The effects of curcumin and capsaicin on serum T-kininogen I level could be either direct or indirect. Curcumin is reported to directly bind to several proteins of varying structures [40]. We have previously demonstrated that curcumin binds to serum albumin [41]. Biophysical studies of curcumin and a newly identified thiol protease inhibitor which belongs to the same class of proteins as T-kininogens, suggests that curcumin may alter the conformation of thiol protease inhibitors [42]. Similar binding studies will be required to detect any direct binding effects of curcumin and capsaicin to the T-kininogen I protein, which may subsequently be degraded. Indirect effects of curcumin and capsaicin could be due to the upstream effects of curcumin and capsaicin on other tissues that influence the serum T-kininogen levels. During the acute phase inflammatory response, an increase in plasma T-kininogen protein level is facilitated by the upregulation of the transcription of T-kininogen genes in the liver [43], [44]. The liver is also prone to an increased lipid peroxidation index during an acute phase response, a process that is ameliorated by dietary administration of curcumin or capsaicin [17], [18], [45], [46]. It is therefore possible that the increase in plasma T-kininogen is an indirect result of the overall ability of these compounds to enhance liver function during both acute and chronic inflammatory responses and thereby facilitate the efficient transcription of the T-kininogen gene. Regardless of the underlying mechanism of action, the present study provides the basis to further explore T-kininogen I as a molecule linked to the significant anti-inflammatory effects of curcumin and capsaicin under the acute phase inflammatory condition.
